# Mobile Device Use While Driving — United States and Seven European Countries, 2011

**Published:** 2013-03-15

**Authors:** Rebecca B. Naumann, Ann M. Dellinger

**Affiliations:** Div of Unintentional Injury Prevention, National Center for Injury Prevention and Control, CDC

Road traffic crashes are a global public health problem, contributing to an estimated 1.3 million deaths annually (1). Known risk factors for road traffic crashes and related injuries and deaths include speed, alcohol, nonuse of restraints, and nonuse of helmets. More recently, driver distraction has become an emerging concern (2). To assess the prevalence of mobile device use while driving in Belgium, France, Germany, the Netherlands, Portugal, Spain, the United Kingdom (UK), and the United States, CDC analyzed data from the 2011 EuroPNStyles and HealthStyles surveys. Prevalence estimates for self-reported talking on a cell phone while driving and reading or sending text or e-mail messages while driving were calculated. This report describes the results of that analysis, which indicated that, among drivers ages 18–64 years, the prevalence of talking on a cell phone while driving at least once in the past 30 days ranged from 21% in the UK to 69% in the United States, and the prevalence of drivers who had read or sent text or e-mail messages while driving at least once in the past 30 days ranged from 15% in Spain to 31% in Portugal and the United States. Lessons learned from successful road safety efforts aimed at reducing other risky driving behaviors, such as seat belt nonuse and alcohol-impaired driving, could be helpful to the United States and other countries in addressing this issue (2,3). Strategies such as legislation combined with high-visibility enforcement and public education campaigns deserve further research to determine their effectiveness in reducing mobile device use while driving. Additionally, the role of emerging vehicle and mobile communication technologies in reducing distracted driving–related crashes should be explored.

HealthStyles and EuroPNStyles are online surveys designed by Porter Novelli (Washington, DC), a worldwide social marketing and public relations firm, and conducted among persons aged ≥18 years to examine health-related attitudes and behaviors. The HealthStyles data analyzed in this study were collected in the 2011 fall HealthStyles survey, conducted in the United States during September 30–October 5, 2011. The fall HealthStyles survey was sent to a random sample of panelists who had completed the 2011 spring HealthStyles survey. The spring HealthStyles survey was drawn from a panel containing 50,000 persons randomly selected through probability-based sampling to be representative of the noninstitutionalized U.S. civilian population; 14,598 panelists were selected to participate in the spring HealthStyles survey, and 8,110 panelists completed the survey (response rate: 56%). The fall HealthStyles survey was sent to 5,315 of the persons who had completed the spring HealthStyles survey; 3,696 (70%) completed the fall HealthStyles survey. Respondents who completed the survey received reward points (worth approximately $10) and were eligible to win a prize through a monthly sweepstakes (prizes generally were worth less than $500). HealthStyles survey data were weighted to match U.S. Current Population Survey proportions for the following nine characteristics: sex, age, annual household income, race/ethnicity, household size, education, U.S. Census region, metro status (i.e., residence in a metropolitan statistical area [MSA] versus a non-MSA), and prior Internet access.

The EuroPNStyles survey was conducted in July 2011 in Belgium, France, Germany, the Netherlands, Portugal, Spain, and the UK. The sample was randomly drawn from Synovate’s Global Opinion Panel, recruited via Synovate partnerships with select websites, portals, and Internet service providers in Belgium, France, Germany, the Netherlands, Spain, and the UK. In Portugal, the sample was randomly drawn from the Global Market Insite’s Panel. Panelists were selected to match each country’s census proportions for age and sex, and quotas were set to reach 1,700 adults in all countries except for Spain and Portugal, where quotas were set to 850 adults. The survey’s response rate in 2011 was 34%, with 10,338 persons completing the survey. Respondents received reward points for completing the survey, and the final data were weighted by age and sex to match each country’s census proportions.

In both surveys, respondents were asked if they had driven in the past 30 days. If they had, respondents were then asked, “In the past 30 days, how often have you talked on your cell phone while you were driving?” and “In the past 30 days, how often have you read or sent a text message or e-mail while you were driving?” Response choices were “never,” “just once,” “rarely,” “fairly often,” and “regularly.” Weighted percentages and corresponding 95% confidence intervals (CIs) for those who had talked on their cell phone while driving at least once (defined as those who responded “regularly,” “fairly often,” “rarely,” or “just once”) and for those who “never” talked on their cell phone while driving were calculated by country, age group, and sex. Similar percentages were calculated for reading or sending text or e-mail messages while driving. Additionally, weighted percentages of those who engaged in these behaviors “regularly” or “fairly often” were calculated and were included as a subset of those who engaged in these behaviors at least once in the past 30 days (Figures 1 and 2).

What is already known on this topic?

Road traffic crashes are a global public health problem, contributing to an estimated 1.3 million deaths annually, and mobile device use while driving has become an emerging concern.

What is added by this report?

In 2011, online surveys of drivers aged 18–64 years revealed that the percentage of those who reported that they had talked on their cell phone while driving ranged from 21% in the United Kingdom to 69% in the United States, and the percentage of those who reported that they had read or sent text or e-mail messages while driving ranged from 15% in Spain to 31% in Portugal and the United States.

What are the implications for public health practice?

To address the problem of mobile device use while driving, countries could consider examining the use of road traffic injury prevention strategies (e.g., legislation combined with high-visibility enforcement by police officers) that have been successful in reducing the prevalence of other road safety risk factors (e.g., alcohol-impaired driving and seat belt nonuse). Additionally, the effectiveness of emerging vehicle and mobile communication technologies should be studied to assess their role in reducing crashes related to distracted driving.

In 2011, more than two thirds (68.7% [CI = 66.4%–71.0%]) of U.S. adult drivers aged 18–64 years reported they had talked on their cell phone while driving at least once in the past 30 days (Figure 1). In Europe, percentages ranged from 20.5% in the UK (CI = 17.7%–23.3%) to 59.4% in Portugal (CI = 54.6%–64.2%). Additionally, 31.2% (CI = 29.0%–33.5%) of U.S. drivers aged 18–64 years reported that they had read or sent text or e-mail messages while driving at least once in the past 30 days (Figure 2). In Europe, percentages ranged from 15.1% (CI = 12.3%–17.9%) in Spain to 31.3% (CI = 27.0%–35.5%) in Portugal.

In the United States, few differences by sex were observed (Figure 3). A significantly larger percentage of both men and women aged 25–44 years reported talking on a cell phone while driving compared with those aged 55–64 years, and a significantly larger percentage of men and women aged 18–34 years reported that they had read or sent text or e-mail messages while driving compared with those aged 45–64 years.

## Editorial Note

This report provides new information on the prevalence of self-reported mobile device use while driving in the United States and seven European countries. Although studies have estimated the prevalence of these behaviors in individual countries, question wording and methods vary, making comparisons difficult. This study used identical questions (with the exception of minor differences resulting from translation into multiple languages) and similar survey methods to examine differences in the prevalence of mobile device use while driving in the eight countries.

The estimates of talking on a cell phone while driving in the United States are consistent with previous research (4–6). In 2010, the AAA Foundation for Traffic Safety conducted a nationally representative telephone survey and similarly found that 69% of drivers aged ≥16 years had used a cell phone while driving, and 24% had texted while driving in the past 30 days (4). Similar estimates also have been reported from surveys carried out by the National Highway Traffic Safety Administration and the Insurance Institute for Highway Safety (5,6). In Europe, recent national estimates of these behaviors are less common. However, a 2003 nationally representative survey in France found that 33% of adults aged ≥18 years reported using a cell phone while driving, whereas the study described in this report indicated that approximately 40% of persons aged 18–64 years in France talk on their cell phones while driving (7). The small difference might be explained by the increased use of cell phones over time and differences in the age groups surveyed.

Several studies support the finding that a greater proportion of younger drivers talk and text while driving compared with older drivers (5–7). Strategies have been aimed specifically at teens and new drivers to try to reduce mobile device use while driving. As of February 2013, a total of 33 U.S. states and the District of Columbia had laws restricting at least some teens or new drivers from using electronic devices while driving. However, these laws alone have not yet proven effective at decreasing these behaviors among young drivers (8).

Additional strategies that have been applied to reduce mobile device use while driving in the United States and other countries include law enforcement efforts, communications campaigns, vehicle and cell phone technological advances, legislation, and education (2). Evaluation data for many of these strategies is both lacking and needed. A few studies have examined the effects of cell phone use laws on the general population and have indicated that laws might be effective in decreasing certain types of cell phone use (e.g., hand-held use), particularly when combined with high-visibility enforcement by police officers (9). However, these laws have not yet been shown to result in decreased crash rates.

The findings in this report are subject to several limitations. First, HealthStyles and EuroPNStyles survey respondents might not be representative of each of the eight country populations because the sampling approaches used were not completely random. However, comparisons of HealthStyles survey responses to those of the Behavioral Risk Factor Surveillance System, a survey which randomly selects persons through probability-based sampling, have shown similar results for various health behavior and disease–related questions in the United States (10). Second, although the HealthStyles sample was not dependent on computer and Internet access (because households that were selected to participate were provided with a laptop computer and access to the Internet if needed), this was not the case for the EuroPNStyles sample, which might affect the representativeness of the estimates in these countries. Third, the findings might be subject to nonresponse bias. If nonresponders were significantly different than responders in their mobile device use while driving behaviors or likelihood of reporting such behaviors, results would be biased. Fourth, the findings might be subject to social-desirability bias; because mobile device use while driving is illegal in many of these countries and often viewed unfavorably, respondents might underreport this behavior, potentially resulting in low estimates. Fifth, because the survey did not ask participants about cell phone ownership and cell phone capabilities (e.g., texting capabilities), some of those responding “never” to these questions might include those that do not have a cell phone or do not have texting capabilities. However, because this study covered persons aged 18–64 years in the United States and Europe, the percentage of those who do not own a cell phone would be expected to be small. Sixth, because prevalence estimates are based on self-reported estimates of mobile device use while driving in the past 30 days, estimates might be affected by recall bias. Finally, this study population was restricted to drivers aged 18–64 years; therefore, prevalence estimates are not representative of the entire driving population in these countries.

Mobile device use while driving is a prevalent behavior in the United States and several countries in Europe. This study revealed a large range in the prevalence of these behaviors, particularly for estimates of talking on a cell phone while driving. It is unlikely that differences in the prevalence of mobile device use while driving between countries are attributable to differing proportions of persons owning mobile devices in these countries, given that mobile markets in developed countries are similarly saturated. It is also unlikely that differences in cell phone use laws fully explain prevalence differences. While U.S. states differ in their cell phone use laws, nearly all European countries have hand-held bans in place, yet there is still a large variation in European estimates. Further research is needed to explore other factors that might help explain these differences, such as differences in strategies (e.g., enforcement and public education campaigns) applied to try to reduce these behaviors and cultural differences regarding the acceptability of these behaviors.

Many countries have made substantial improvements in reducing other risky driving behaviors, such as seat belt nonuse and alcohol-impaired driving, through a combination of legislation, sustained and highly visible enforcement, and ongoing public education campaigns to increase awareness of the risks and penalties associated with disobeying traffic laws (2,3). Countries could consider exploring the effectiveness of applying similar approaches to the problem of mobile device use while driving. Additionally, the effectiveness of emerging vehicle and mobile communication technologies (e.g., advanced crash warning and driver-monitoring technologies or applications that temporarily disable mobile devices while a vehicle is in motion) should be studied to assess their role in reducing crashes related to distracted driving.

## Figures and Tables

**FIGURE 1:**
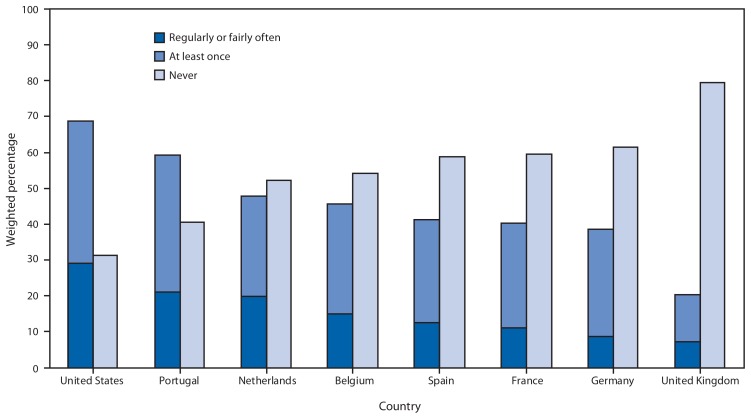
Weighted percentage of adults aged 18–64 years who reported that they had talked on their cell phone while driving regularly or fairly often, at least once, or never in the past 30 days,^*^ by country — HealthStyles and EuroPNStyles, 2011 ^*^Respondents were asked, “In the past 30 days, how often have you talked on your cell phone while you were driving?” Response choices were “never,” “just once,” “rarely,” “fairly often,” and “regularly.” Percentages of those who engaged “at least once” were defined as those who responded “just once,” “rarely,” “fairly often,” or “regularly.” Percentages of those who responded “regularly” or “fairly often” are shown as a subset of “at least once.”

**FIGURE 2:**
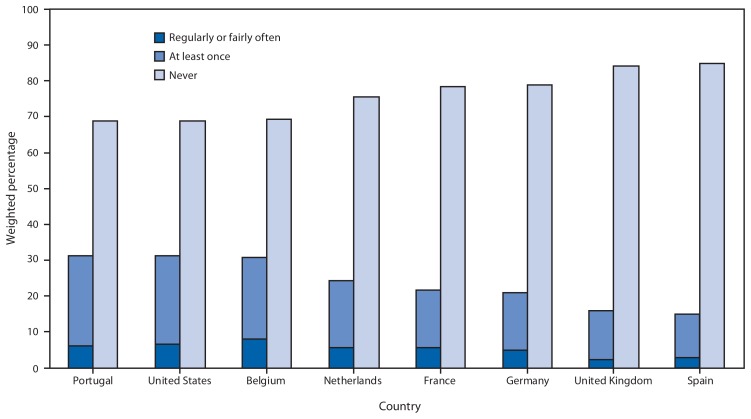
Weighted percentage of adults aged 18–64 years who reported that they had read or sent text or e-mail messages while driving regularly or fairly often, at least once, or never in the past 30 days,^*^ by country, HealthStyles and EuroPNStyles, 2011 ^*^ Respondents were asked, “In the past 30 days, how often have you read or sent a text message or e-mail while you were driving?” Response choices were “never,” “just once,” “rarely,” “fairly often,” and “regularly.” Percentages of those who engaged “at least once” were defined as those who responded “just once,” “rarely,” “fairly often,” or “regularly.” Percentages of those who responded “regularly” or “fairly often” are shown as a subset of “at least once.”

**FIGURE 3:**
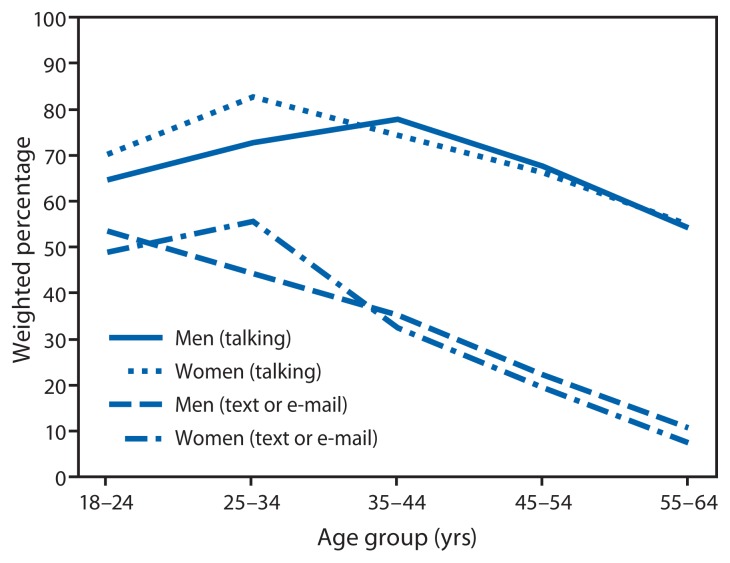
Weighted percentage of adults aged 18–64 years who reported that they had talked on their cell phone while driving at least once and read or sent text or e-mail messages while driving at least once in the past 30 days,^*^ by sex and age group — United States, HealthStyles, 2011 ^*^ Respondents were asked, “In the past 30 days, how often have you talked on your cell phone while you were driving?” and “In the past 30 days, how often have you read or sent a text message or e-mail while you were driving?” Response choices were “never,” “just once,” “rarely,” “fairly often,” and “regularly.” Percentages of those who engaged “at least once” were defined as those who responded “just once,” “rarely,” “fairly often,” or “regularly.”
